# Ultrasound Triggering of Liposomal Nanodrugs for Cancer Therapy: A Review

**DOI:** 10.3390/nano12173051

**Published:** 2022-09-02

**Authors:** Wafa N. Bahutair, Waad H. Abuwatfa, Ghaleb A. Husseini

**Affiliations:** 1 Department of Chemical Engineering, College of Engineering, American University of Sharjah, Sharjah P.O. Box. 26666, United Arab Emirates; 2 Materials Science and Engineering Program, College of Arts and Sciences, American University of Sharjah, Sharjah P.O. Box. 26666, United Arab Emirates

**Keywords:** liposomes, passive targeting, active targeting, receptor-mediated endocytosis, ultrasound, triggered release

## Abstract

Efficient conventional chemotherapy is limited by its nonspecific nature, which causes severe systemic toxicity that can lead to patient discomfort and low therapeutic efficacy. The emergence of smart drug delivery systems (SDDSs) utilizing nanoparticles as drug nanocarriers has shown great potential in enhancing the targetability of anticancer agents and limiting their side effects. Liposomes are among the most investigated nanoplatforms due to their promising capabilities of encapsulating hydrophilic, lipophilic, and amphiphilic drugs, biocompatibility, physicochemical and biophysical properties. Liposomal nanodrug systems have demonstrated the ability to alter drugs’ biodistribution by sufficiently delivering the entrapped chemotherapeutics at the targeted diseased sites, sparing normal cells from undesired cytotoxic effects. Combining liposomal treatments with ultrasound, as an external drug release triggering modality, has been proven effective in spatially and temporally controlling and stimulating drug release. Therefore, this paper reviews recent literature pertaining to the therapeutic synergy of triggering nanodrugs from liposomes using ultrasound. It also highlights the effects of multiple physical and chemical factors on liposomes’ sonosensetivity, several ultrasound-induced drug release mechanisms, and the efficacy of ultrasound-responsive liposomal systems in cancer therapy. Overall, liposomal nanodrug systems triggered by ultrasound are promising cancer therapy platforms that can potentially alleviate the detriments of conventional cancer treatments.

## 1. Introduction

Cancer annually threatens and claims the lives of millions of people worldwide. In 2020, around 19.3 million cancer cases and about 10 million cancer-related deaths were reported [1]. The severity of this heterogenous disease has prompted the need to develop various treatments. Currently, the most conventionally utilized cancer therapies are surgery, radiation, immunotherapy, and chemotherapy [2]. The latter, however, is the most common modality to treat metastatic cancers. This is due to the chemotherapeutics’ ability to circulate in the bloodstream and distribute into the entire body, thereby targeting cancer tissues at more than one site. At the same time, a significant share of the drug attacks rapidly growing healthy cells [3,4]. The subsequent damage of normal cells is what leads to the well-known side effects of conventional chemotherapy, including hair loss, nausea, fatigue, and myocardial infarction, alongside their psychological ramifications on the patient [5,6]. This systemic toxicity may reduce the treatment efficiency by restricting the administered chemotherapeutic doses prescribed to the patient. Consequently, tumors may relapse or develop multi-drug resistance (MDR), a phenomenon where cancer cells can survive in the presence of anticancer drugs [7].

Besides deteriorating cancer patients’ quality of life, another major problem is also associated with traditional chemotherapy, which is the drug’s low availability at the tumor site. Such a drawback is the result of a number of factors—one of which is that many chemotherapeutics exhibit low blood solubility and might not be able to avoid enzymes’ attacks while in blood circulation [8,9]. Furthermore, a significant amount of the chemotherapy dosage is lost to healthy tissues rather than being exclusively delivered to cancer cells. Even upon arrival to destination cells, the tumor’s high interstitial pressure and abundant stroma may hinder the drug’s penetration deep through the tumor tissues [7]. On top of that, many antineoplastic agents show poor cellular uptake, leading to low intracellular drug concentration [8]. Since the treatment efficiency strongly depends on the success of the chemotherapy drugs to abundantly reach the tumor [10], the addressed obstacles should be overcome to achieve better therapeutic outcomes. 

In light of what preceded, new technologies are needed to minimize or eliminate the side effects of conventional chemotherapy and enhance its efficacy. Further understanding of cancer pathology and the biological disparities between cancer and healthy tissues have led to the development of a new treatment modality that can achieve the above purposes [11]. Such a modality harnesses nanoparticles as Smart Drug Delivery Systems (SDDSs) that can carry the chemotherapy dosage and deliver it specifically to its targets. This is followed by a sustained release of the drug from the nanocarrier into the cancer cell. The drug release can be regulated by sensitizing the nanocarriers to certain stimuli so that they remain stable until a stimulus is applied to the desired diseased areas [12]. Therefore, such nanosystems can alter both the biodistribution and pharmacokinetics of chemotherapeutics, resulting in lower systemic toxicities and higher therapeutic indices [13]. 

A wide range of materials is used to design chemotherapy nanocarriers, including polymers (e.g., dendrimers and micelles), lipids (e.g., liposomes and niosomes), and inorganic materials (e.g., carbon nanotubes, metallic nanoparticles, and quantum dots) [14]. Several stimulus-responsive nanoplatforms are currently under study; however, ultrasound-responsive liposomes have gained increasing attention. Liposomes have been shown to be effective in improving drug tolerance and reducing systemic toxicity [15,16]. With liposomes’ remarkable physical and chemical properties, as well as ultrasound ease of control and non-invasiveness, such a nanosystem appears promising as a treatment for cancer. Therefore, this article reviews the use of ultrasound to induce drug release from liposomal systems and presents some relevant *in vitro* and *in vivo* work.

## 2. Liposomes as Nanocarriers in SDDSs

The non-selective nature of the traditional cancer therapy platforms leads to the limited efficiency of the drug, poor drug biodistribution, and high toxicity to healthy tissues, resulting in serious side effects. These shortcomings can be overcome by temporally and spatially controlling the delivery of the drugs. Controlled SDDSs deliver the therapeutic agents of interest specifically to the diseased tissues, thereby reducing their toxic effects on the rest of the body. SDDSs also increase the drug’s blood circulation time by minimizing its clearance rate and rapid degradation, thereby lowering the required drug doses, and enhancing its efficacy [17]. 

Targeted therapy can be achieved by sequestering anticancer agents inside nano-sized vehicles (nanoparticles), ranging in diameter from 1 to 100 nm [18]. In contrast to macroparticles, nanoscale sizes impose numerous unique characteristics, such as enhanced optical, magnetic, and electrical properties [19]. In general, all nanoparticles share a high surface area to volume ratio, enhanced reactivity, and improved drug capacity, thereby enhancing drug bioavailability in the tumor and reducing dosage frequency [20].

**Figure 1:**
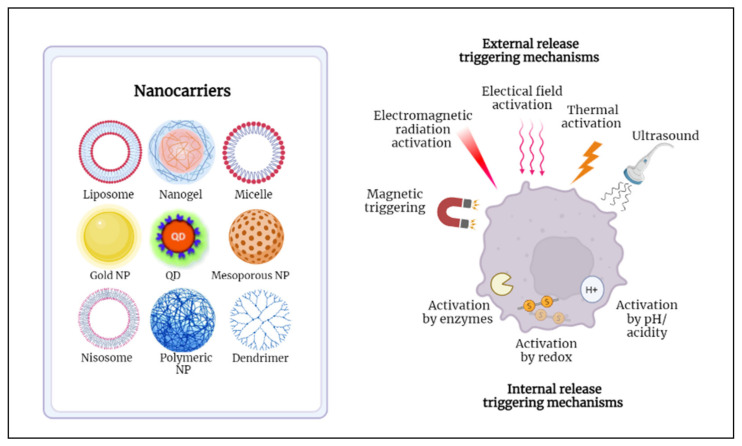
Presentation of the different nanocarriers and triggering mechanisms used in developing SDDSs.

Nanoparticles that are used to transport drugs to specific sites are referred to as nanocarriers. Conventional nanocarriers lack the ability to deliver the right drug concentration to target cells and exhibit slow drug release profiles. In order to make such nanosystems “smart”, they must be properly engineered to accumulate preferentially at cancer cells, increase intracellular cytotoxicity, and provide a fast and controlled release of their encapsulated cargo. To achieve such properties, nanocarriers surfaces should be modified with hydrophilic molecules to evade the cleanse by the immune system and with targeting ligands to promote cellular uptake by cancer cells [21]. Additionally, to induce the entrapped drug release, several stimuli can be used, and they can be either endogenous (e.g., pH, enzymes) or exogenous (e.g., temperature, radiation, ultrasound) [22]. Different types of nanocarriers and triggering modalities are illustrated in Figure 1.

Various nanoparticles (NPs) have been investigated as SDDSs that can efficiently deliver the entrapped drugs to their targets. NPs can be classified into inorganic and organic. Inorganic NPs include gold NPs, quantum dots, and ceramic NPs, whereas organic NPs include dendrimers, micelles, and liposomes [23]. 

**Figure 2:**
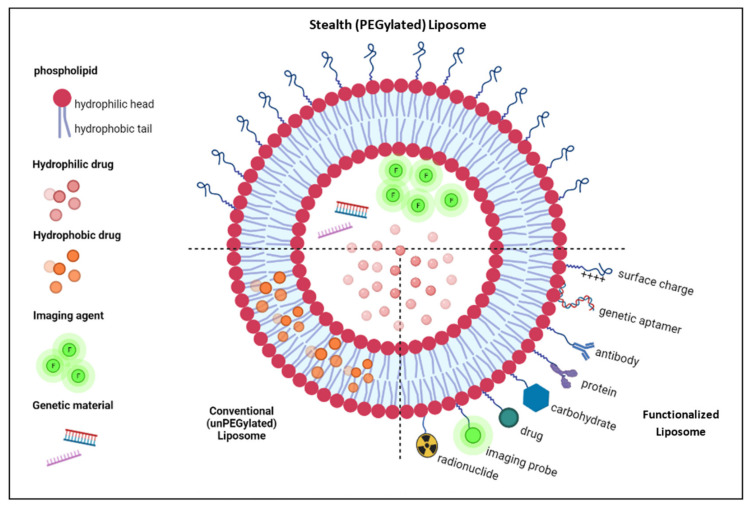
Possible loading, functionalization, and surface engineering schemes of liposomes.

Liposomes are nanosized vesicles composed of phospholipid bilayer membrane entrapping an aqueous compartment. Their lipid bilayer resembles the cellular membrane. That is, it is mainly composed of phospholipids (a hydrophilic head attached to two apolar hydrophobic tails), cholesterol, and non-toxic surfactants. Liposomes form spontaneously when amphipathic phospholipids are hydrated. When dispersed in an aqueous solution, the polar heads of these molecules (composed of phosphate groups) tend to interact with water molecules by hydrogen bonds and polar interactions. On the other hand, the hydrophobic fatty acid tails repel water and instead attract each other to minimize their contact with water molecules. The Van der Waals interactions between these tails keep them closely packed and stable. This results in the formation of a spheroidal bilayer arrangement (liposome) in which hydrophobic tails from each layer are directed opposite to each other, thereby constituting the inner compartment of the lipid bilayer membrane, while the hydrophilic heads are pointed toward the exterior and interior aqueous solutions, hence forming the outer and inner surfaces of the liposomes (Figure 2) [23,24,25]. 

Since they were first discovered, liposomes have shown great potential as SDDS due to their unique biological and physicochemical properties—one of which, owing to their dual nature, is their ability to immobilize both hydrophilic drugs in their aqueous core and hydrophobic drugs within the lipid bilayer [26]. In addition, and since they are composed of the same materials as the cellular membrane, they are highly biocompatible, biodegradable, and non-toxic to the body. Moreover, liposomes have shown high drug loading capacity and high solubility in water (thus blood). Furthermore, their surface can be modified to enhance their stealth properties (hence prolonging their body lifespan) and promote active targeting [24]. Due to the stated properties, liposomes have demonstrated huge success as drug delivery systems, as some are already in the market (see Table 1), others are pending approval, and some are in clinical trials. One of the successful liposomal formulations for cancer treatment that are currently on the market is Doxil (a PEGylated doxorubicin liposomal formulation) as it was approved by the United States of America’s Food and Drug Administration (FDA) in 1995 [27].

**Figure 3:**
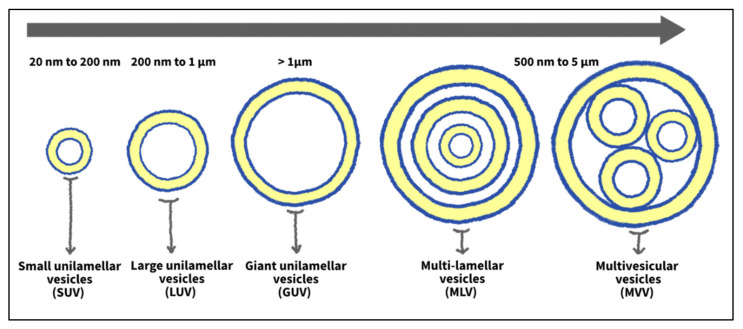
Classification of liposomes based on size and lamellarity.

Liposomes can be classified based on size and the number of lipid bilayers they possess (structure/lamellarity). Liposomes with several concentric phospholipid-bilayer spheres separated by fluid compartments are designated as multilamellar vesicles (MLVs) (like an onion structure). On the other hand, liposomes with a single lipid bilayer enclosing a fluid core are referred to as unilamellar vesicles (ULVs). The latter can be further subdivided, based on size, into giant unilamellar vesicles (GUVs), large unilamellar vesicles (LUVs), and small unilamellar vesicles (SUVs). Typically, the multilamellar structure is generated when several ULVs are formed around other smaller ones [25,33]. Another type of vesicles exists, which is multivesicular vesicles (MVVs). The MVVs are composed of non-concentric bilayers clustered in one large vesicle. Figure 3 illustrates the classification of liposomes according to size and lamellarity. Another way to categorize liposomes is based on their composition. They can be classified into conventional, fusogenic, pH-sensitive, cationic, long-circulating, and immuno-liposomes [34].

Liposomes face several immunogenic barriers in the body that impede them from sufficiently accumulating at the tumor site. One such obstacle is the process of opsonization—the process by which serum proteins (called opsonin proteins) are adsorbed by the surface of foreign bodies to allow phagocytes (from the reticuloendothelial system, RES) to recognize and eliminate them. As soon as liposomes are administered into the bloodstream, opsonin proteins bind to their surfaces, rendering them susceptible to phagocyte attack. Phagocytes ingest the liposomes and remove them from the blood circulation by depositing them in the liver or spleen, where they are removed from the body. This results in short blood circulation times of the liposomes when used *in vivo*, lowering their accumulation at intended sites [35]. 

To avoid opsonization, liposomes are coated with hydrophilic molecules that reduce the adsorption of opsonin proteins on their surface, thus shielding them from RES recognition. The most commonly used molecule for this purpose is polyethylene glycol (PEG). The process by which liposomes are conjugated with PEG is named PEGylation, whereas the modified liposomes with enhanced stealth properties are designated as “stealth liposomes.” PEG molecules form a protective hydrophilic layer around the liposome, which repels opsonins away from the liposomal surface. This, in turn, reduces the uptake of the liposomes by phagocytes, which prolongs their circulation times from minutes to more than a day. Consequently, enhanced localization of liposomes at the tumor interstitium is achieved, thus resulting in better therapeutic efficiency [35,36]. Besides preventing the interaction of the liposomes with the macromolecules in the body, PEGylation also offers high colloidal stability by enhancing inter-particle steric repulsion. In addition, targeting molecules can be incorporated with the terminal functional group of PEG chains to induce the internalization of the liposomes by cancer cells via active targeting [37].

## 3. Tissue-Specific Targeting Mechanisms: Passive and Active Targeting

Generally, the ability of nanocarriers to specifically target cancer relies on the anatomical and physiological differences between healthy and cancerous tissues. A key difference is the enhanced permeability and retention (EPR) features that characterize tumors. These features are critical for targeting cancer cells, as they allow specifically designed nanocarriers to escape the defected vasculature of cancer tissues and ultimately accumulate at the diseased locations. This method of targeting cancer is referred to as passive targeting which occurs spontaneously [26]. The enhanced permeability effect mainly stems from the differences in the angiogenesis process between cancer and normal tissues. Tumor angiogenesis occurs at significantly faster rates than in normal tissues, which results in the formation of blood vessels with irregular discontinued epithelium that lacks a basal membrane. This leaky nature of the tumor vasculature permits nanoparticles to permeate effortlessly through the tumor capillaries to the cancer interstitium. On the contrary, normal tissues are surrounded by capillaries with tight junctions that prevent nanocarriers from penetrating and accumulating in their vicinity, hence minimizing the drug toxicity to healthy cells [26,38]. On the other hand, the enhanced retention effect results from the lack of a functional lymphatic drainage system around the tumor. Lymphatic vessels are responsible for removing excess water or any foreign bodies from the interstitial fluid bathing around the cells. However, as the tumor grows, it compresses these lymphatic vessels and eventually damages most of them. This defective lymphatic system prolongs the retention time of the nanocarriers, thereby preferentially accumulating them in tumor tissues [26,38,39].

It is important to note that several immunogenic barriers hinder the delivery of nanocarriers to the tumor site—one of which is the reticuloendothelial system (RES) in the liver and spleen, whose function is to relieve the body of old cells and foreign bodies. These organs consist of dilated irregular microvasculature structures with discontinuous porous epithelium (sinusoids) lined with phagocytic cells [39]. When normal blood cells and small particles pass through the sinusoids, they permeate through the gaps in the walls, while large particles (including old or abnormal cells and large foreign bodies) fail to escape the sinusoids; thus, they get attacked and cleansed by the phagocytic cells [40]. In this regard, an ideal SDDS should be small enough to escape the capture by the phagocytes in the liver and spleen, but it should be large enough to only pass through the cancer vasculature and not through the healthy ones. The size of the gaps in the sinusoids varies from 150 to 200 nm [39]. In contrast to healthy tissues’ vasculature, whose junctions are of the size of 5–10 nm, the size of gaps in leaky tumor vasculature ranges from 100–780 nm [41]. Based on the literature, the size of the nanocarriers used for cancer treatment should preferably be in the range of 70–200 nm to effectively (large amounts by escaping the RES) and selectively (specific sites) target cancer tissues [42].

The efficient localization of the drug carriers in the tumor interstitium is the result of the EPR effect; however, an efficient treatment also entails increasing the uptake of these accumulated nanocarriers by cancer cells. Passive targeting (based on the EPR effect) cannot promote such cellular uptake, which brings the need for a second type of targeting, i.e., active targeting [43]. Cancer cells often overexpress specific receptors on their membranes, unlike normal cells. This disparity in normal and diseased cells’ metabolisms is advantageous in delivering the drug to the tumor only. This is achieved by conjugating certain molecules, referred to as ligands or moieties, that have a high affinity toward the distinct cancer receptors to the nanocarrier’s surface in a process called surface functionalization. Such modification promotes the binding between the moieties and the cancer receptors (similar to a lock and key mechanism), which, in turn, enhances the cellular uptake of the nanocarrier [26,44,45]. When the loaded functionalized nanocarriers accumulate at the tumor site via the EPR effect, the ligands on their surface bind with the cancer receptors inducing a process referred to as receptor-mediated endocytosis. Receptor-mediated endocytosis involves the cell membrane invaginating and engulfing specific material into the cell upon binding to the cell membrane receptors. These specific receptor–ligand interactions enhance nanoparticle internalization and facilitate the next step in the treatment, that is, the drug release inside the cell [44]. 

The choice of the targeting moiety depends on the receptor or antigen that is selectively expressed or overexpressed on cancer cells relative to healthy ones. However, because only a few tumors have uniquely specific antigens or receptors, researchers have been mainly investigating liposomal formulations that target antigens or receptors overexpressed by cancer cells [13]. Another criterion that contributes to selecting the proper moieties is their ability to target internalizing antigens or receptors to induce receptor-mediated endocytosis. The latter internalizes the targeting liposomes into the acidic interior of the cell. After that, the liposomes are degraded by lysosomal and endosomal enzymes, which release their therapeutic content into the cytoplasm. Higher concentrations of the free drug inside the target cell are desired as they enhance the therapeutic outcomes [13]. Other aspects that should be considered while choosing the targeting ligands include careful selection of the bonds used to conjugate the ligands to the liposomes’ surface, which should be non-toxic and stable. In addition, coupling the liposomes with moieties should not negatively impact the drug loading capacity nor the release rate. It is essential to maintain the biological properties of the ligands (such as their target recognition and binding affinity towards cancer receptors) after the conjugation with the liposomes. Furthermore, the targeted liposomes should have long half-lives in the body to sufficiently accumulate and interact with the tumor cells, and the ligand density per nanoparticle should be optimized to prevent the interaction with serum proteins and recognition by the RES [13,46,47]. A study by Sapra et al. [48] investigated active targeting by comparing the therapeutic effects of two populations of doxorubicin (DOX)-encapsulated liposomes on the human B-lymphoma (Namalwa) cell population. These cells overexpress two different epitopes; one is internalizing (CD19), while the other is not (CD20). Moreover, one of the liposomal samples was conjugated with anti-CD19, whereas the other was coupled with anti-CD20. The experiments showed that anti-CD19-targeted DOX-loaded liposomes resulted in better therapeutic outcomes compared to the liposomes targeting the non-internalizing antigen (CD20). Other experiments carried out in some tumor models have shown that ligand-targeted liposomes (LTLs), targeting internalizing receptors, achieved an increased therapeutic activity as compared to non-targeted liposomes. However, in other tumor models, the utilized LTLs did not enhance the therapeutic efficiency, which was attributed to the lack of internalization of these liposomal formulations by cancer cells [13].

**Figure 4:**
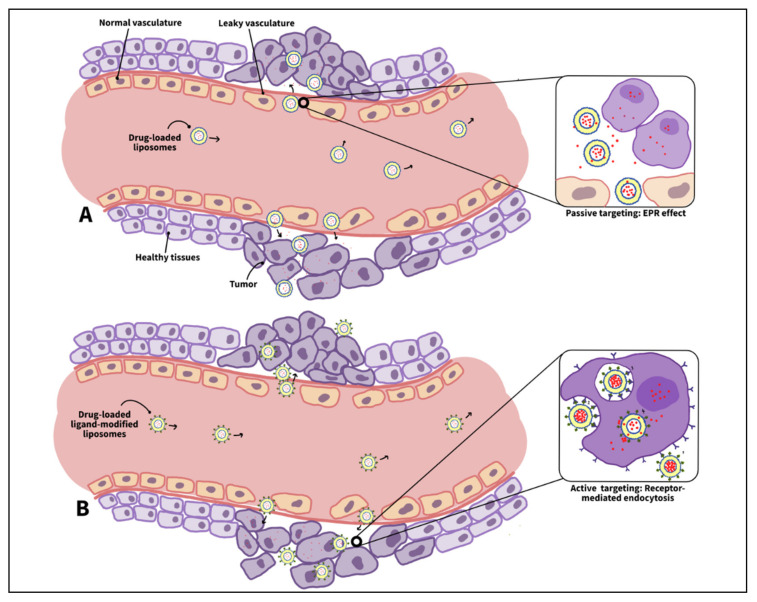
(**A**) Passive targeting. Liposomes leave the blood circulation and accumulate in the tumor vicinity via the EPR effect, which relies on the tumor’s defective vasculature and lymphatic drainage system. Then, the drug diffuses out of the liposomes and enters the cancer cells; (**B**) active targeting. After passive targeting, cancer cells internalize the liposomes via receptor-mediated endocytosis. The latter is induced by the ligand–receptor interactions between cancer cells and liposomes.

While both passive and active targeting have demonstrated several advantages in cancer therapy; they each suffer from some practical and technical limitations. Passive targeting relies on the tumor’s defective vasculature to increase drug concentration in cancer tissues, however; tumors are heterogenous structures that show significant differences in their microenvironment. Some tumors may have tight epithelium, high interstitial fluid pressure, or high tumor cell density, making it difficult for even small-sized nanoparticles to diffuse into the tumor vicinity. Such tumor architecture may render passive targeting ineffective in delivering sufficient drug payloads to diseased tissues [49]. Unlike passive targeting, active targeting does not control the drug biodistribution. Instead, it triggers the NPs’ internalization by receptor-mediated endocytosis upon binding to cancer receptor sites. In addition, active targeting can help overcome the brain–blood barrier (BBB) and the MDR in tumors by bypassing P-glycoprotein-mediated drug efflux [50]. Several drawbacks are associated with this targeting strategy. For instance, active targeting can achieve high selectivity when tested *in vitro* but not necessarily *in vivo.* In addition, conjugating the moieties to the nanocarriers might affect their binding affinity to cancer receptors. Furthermore, conjugating targeting ligands to the nanocarriers’ surface might compromise their stealth properties [51]. Table 2 compares the advantages and disadvantages of passive and active targeting approaches, and Figure 4 schematically illustrates the two targeting mechanisms.

## 4. Ultrasound as an External Triggering Modality

Targeting the tumor cells is the first step in delivering the drug precisely to the diseased area. This should be followed by controlling the drug’s release kinetics in tumors. Different mechanisms have been suggested to achieve such a purpose, and they are classified into internal and external triggers. Internal stimuli rely on the pathological changes in the tumor microenvironment, such as lower pH values, higher temperatures, or overexpression of several proteolytic enzymes. On the other hand, external triggers involve external factors such as heat, magnetic field, light, or ultrasound. Liposomes are designed to be sensitive to at least one of these stimuli to maintain their structure while circulating in the bloodstream but disintegrate and release the encapsulated drug when exposed to such stimulus [52]. This review discusses the use of ultrasound to trigger the drug release from liposomes.

Ultrasound is sound waves with frequencies higher than the upper limit of human hearing (≥20 kHz). As a mechanical wave, ultrasound needs a medium, be it solid, liquid, or gas, to transfer its energy from one location to another. As it propagates, it creates alternating cycles of increased and reduced pressure (compression and rarefaction, respectively), which, in turn, cause the molecules of said medium to compress and expand accordingly [53,54]. Ultrasound’s physical nature (actual movement of molecules), ability to transfer energy, non-invasiveness, safety record, and relatively low cost have made it a valuable tool for medical applications, including diagnosis and therapy. For most medical purposes, ultrasound is produced by a transducer containing a piezoelectric crystal. Piezoelectric crystals are special materials that convert electrical signals into mechanical pulses (ultrasound) and vice versa [55]. When the piezoelectric crystal is subjected to voltage, it vibrates, which pushes and pulls the surrounding air molecules, creating an ultrasonic wave. When the generated ultrasound wave hits an object, part of it reflects back to the transducer. It gets received by the piezoelectric crystal, which then converts this mechanical pulse into an electrical signal. The received signals are then translated into images that help diagnose medical conditions [56]. In contrast to diagnosis, using ultrasound for therapeutics does not require a receiver, as sonication is employed only to break an object in the body (e.g., kidney stone) or to increase the cellular membrane’s porosity through sonoporation [57,58,59].

There is an essential difference between therapeutic and diagnostic applications of ultrasound. Diagnostic applications include using ultrasound to gather information about medical conditions without causing biological effects on the body. On the other hand, therapeutic applications require depositing sufficient energy into the body to produce a desired biological effect. Due to this, different medical applications employ different intensities of ultrasound [60]. The intensity or power density of an ultrasonic beam refers to the time rate at which ultrasonic energy is transferred per one unit area, in other words; it is ultrasound’s power divided by the area of the beam $\left(\frac{J}{s.cm^{2}}=\frac{W}{cm^{2}}\right)$, and is given by the following equation:

(1) $$I=\frac{P^{2}}{\rho c}\;$$

where (*I*) is ultrasound intensity, (*P*) is the acoustic pressure, (ρ) is the medium density, and (*c*) is ultrasound velocity through the medium [60]. Low-intensity ultrasound is used for diagnostic purposes such as imaging and flow studies, while high-intensity ultrasound is used for treatments such as physiotherapy, medical therapy, and drug delivery [54,61].

In liposomal targeted therapy, ultrasound can be used for two different applications: (1) liposome preparation and (2) drug release stimulation. As for liposome synthesis, sonication is widely used to downsize multilamellar vesicles (MLVs) to small unilamellar vesicles (SUVs) [62,63,64]. Optimization of liposome size is essential as large-sized liposomes are more rapidly cleared by RES and thus have a shorter blood lifespan compared to small-sized liposomes [65]. For such a purpose, lipid suspensions containing MLVs are usually sonicated using an ultrasonic bath or a probe tip sonicator. Consequently, acoustic energy delaminates the outer layers of MLVs, forming SUVs [66]. On the other hand, in the last few decades, ultrasonic energy has also shown the potential to stimulate drug escape from liposomes by destabilizing their membranes. Ultrasound-induced physical effects, in the propagation medium, are the reason behind this lipid bilayer disruption. These physical effects can be thermal or mechanical. Thermal effects involve generating hyperthermia that can cause the lipid bilayer to transition from solid-ordered phase to liquid-disordered phase, increasing the liposomal permeability and allowing for drug escape. In contrast, mechanical effects include acoustic cavitation resulting from variations in pressure in a fluid medium [67]. 

Ultrasound, like other waves, carries an amount of energy within it. As it travels through tissues, it transfers its energy to the medium particles and sets them into vibratory motion. In an ideal elastic medium, there is no energy loss, as this obtained kinetic energy is not converted into heat. However, within an actual medium, frictional forces exist, affecting the motion of the oscillating molecules. Hence, part of their acquired kinetic energy is transformed into heat and absorbed by the tissues, increasing their temperature while reducing the ultrasonic wave energy [54]. Such hyperthermia is utilized to heat or damage tumor tissues, or/and to melt thermo-sensitive nanocarriers, thus releasing their therapeutic content at a specific location. The lipid bilayer composition of liposomes plays a role in determining their ultrasound sensitivity. The high compositions of thermosensitive compounds within the membrane make the liposomes susceptible to US-induced hyperthermia, thereby releasing their chemotherapeutic payloads [54]. Furthermore, PEG-lipids’ attachment to the liposomal membrane enhances its temperature sensitivity, as reported by Kono et al. [68]. This is hypothesized because of the absorption of acoustic energy by the highly hydrated PEG headgroups [54].

Apart from thermal effects, ultrasound can result in mechanical effects that aid in the faster diffusion of the drug and/or in its release from the nanocarriers at desired sites. Such mechanical effects involve acoustic cavitation resulting from the changes in pressure caused by the propagating ultrasonic wave [26]. 

Acoustic cavitation involves the formation, growth, oscillating, and collapse of gas-filled microbubbles present in a liquid medium due to pressure variations [69]. Stable bubbles might pre-exist in the bulk fluid or be formed during the rarefaction half-cycle of ultrasound. In the latter, the pressure can drop to values below the vapor pressure of the liquid, thus allowing it to be drawn out of the solution as a gas, forming a microbubble [70]. When subjected to ultrasound, these gas-filled cavities can undergo two types of cavitations, i.e., stable and inertial. In stable cavitation, the microbubble oscillates linearly and gently about an equilibrium size (acoustic pressure in stable cavitation is low; acoustic pressure represents the difference between the pressure when ultrasound is applied and without ultrasound). The oscillating surface of the bubble swirls the surrounding liquid, creating what is known as micro-streaming, which generates shear stresses in the fluid around the vibrating gas cavity [54]. 

**Figure 5:**
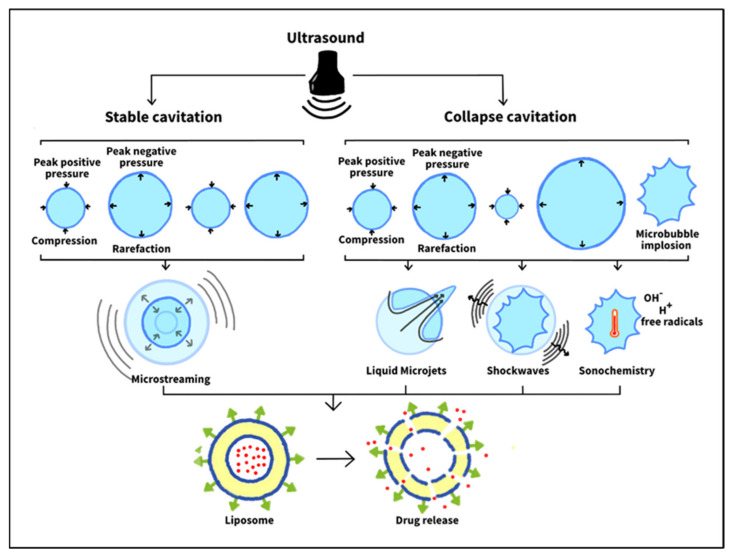
Ultrasound-induced mechanical effects include cavitation, which can be either stable or collapse. In stable cavitation, the microbubble oscillates about an equilibrium size, resulting in microstreaming in the surrounding liquid. On the contrary, in collapse cavitation, the microbubble exceeds its equilibrium size and expands until it implodes. This, in turn, leads to three distinct outcomes: (1) liquid microjets formation, (2) shockwaves, and (3) sonochemistry. All these together can destabilize the liposomal lipid bilayer, releasing the encapsulated cargo.

On the other hand, inertial or collapse cavitation involves unsteady oscillations and rapid growth of the microbubble. Its radius increases until it exceeds its resonant size, after which it grows abruptly and collapses aggressively [71,72]. The bubble collapse spawns smaller bubbles that can expand and then burst. This size increase of the radially oscillating bubble occurs due to rectified diffusion. In this process, during the expansion half cycle, the pressure inside the bubble drops, allowing liquid molecules in the surrounding medium to diffuse into the bubble and vaporize. During the subsequent contraction half cycle, the pressure inside the bubble increases, causing some of the vapor inside to condense and diffuse outwards. The surface area of the bubble in the expansion phase is much larger than this in the contraction phase, allowing more molecules to diffuse into the bubble than out of it. This, in turn, causes the gas cavity’s vapor to increase over time, leading to its rapid growth over a few cycles followed by its collapse [54]. Subsequently, collapse cavitation brings about three potential outcomes: sonochemistry, shockwaves, and liquid microinjections [54,73]. Figure 5 illustrates ultrasound-induced cavitation types and how they contribute to drug release from liposomes.

Sonochemistry is a sudden collapse of the gas cavity resulting in substantial temperatures and pressures within the bubble’s core. When molecules are exposed to such high temperatures (which might reach 5000 K), they fragment and might form free radicals such as reactive oxygen species (ROS). Free radicals are unstable and might react with essential components in the cell, such as its DNA, RNA, or proteins, eventually leading to its death. Such an approach is utilized to damage tumor tissues [73]. 

Another mechanical effect of collapse cavitation is the formation of shockwaves that propagate through the surroundings. These shockwaves can have amplitudes exceeding 10,000 atmospheres (based on the bubble size) and create shear stresses that disrupt the membranes of surrounding cells or vesicles (such as liposomes), rendering them permeable through a process called sonoporation. This, in turn, induces the release of the drug from the liposomal formulations and improves its uptake by the permeable cells. Sonoporation was demonstrated experimentally by Yudina et al. in 2010 [74]. In their *in vitro* study, cell impermeable optical chromophores were added to C6 cells and then exposed to ultrasound. Only the sonicated cells showed high fluorescence, indicating that chromophores diffused faster into the cells through the pores formed by sonoporation. Furthermore, this enhanced cell permeability persisted for 24 h after ultrasound exposure. *In vivo* studies have also proven the sonoporation effect of ultrasound by successfully delivering impermeable macromolecules such as bleomycin to tumor tissues and enhancing the permeability of tumor cells, thereby increasing the uptake of chemotherapeutic drugs [74].

The previous effect (shockwaves) occurs when the bubble collapses in the bulk fluid. However, when inertial cavitation happens on or near a solid surface, another mechanical effect occurs: the formation of liquid microjets. Microjets form in this case because of the asymmetric collapse of a gas-filled bubble near a solid (it collapses symmetrically when surrounded only by liquid). These microjets travel with high velocities that can reach hundreds of meters per second. Hence, when they strike the neighboring solid (cell or nanocarrier), they pierce it. They also can form secondary shockwaves, which, in turn, travel through the tissues and perforate the membranes in their path. The combined effect of microjets and their secondary shockwaves in the treatment of cancer includes inducing the release of drugs from nanocarriers (e.g., liposomes), enhancing cellular uptake of the drug, and causing membrane damage and, perhaps, cell death [73,75]. 

The likelihood of inertial cavitation occurrence in a sonicated liquid medium can be determined by evaluating what is known as the mechanical index (*MI*), which is given by the following formula:

(2) $$MI=\frac{P_{neg}}{\sqrt{f}}\;$$

where $\left(P_{neg}\right)$ is the peak rarefactional pressure (maximal negative pressure) in MPa and $\left(f\right)$ is the frequency of ultrasound in MHz. A mechanical index of 0.7 indicates a high possibility of collapse cavitation in the medium. Values below 0.5 favor stable cavitation, while those more than 0.5 burst the microbubbles [76]. Another factor that influences the possibility of cavitation occurrence is the number of gas cavities available in the medium. The higher the number of gas microbubbles, the higher the likelihood of collapse cavitation. Furthermore, each bubble size has a certain ultrasound intensity threshold, above which transient cavitation starts. Generally, this intensity threshold decreases, as the ultrasound frequency decreases. Thus, collapse cavitation is more likely to happen at lower frequencies of ultrasound [54].

## 5. *In Vitro* and *In Vivo* Studies on Liposomal SDDSs Triggered by Ultrasound

Several studies have shown that ultrasound can induce drug release from liposomes by giving rise to either thermal or mechanical effects. Herein, some *in vitro* and *in vivo* work performed on liposomal drug delivery systems using ultrasound (US) is reviewed. 

Weinstein et al. [77] were the first to synthesize temperature-sensitive liposomes (TSL) in 1980 to treat mice bearing L1210 tumors, implanted in both hind feet. Liposomal methotrexate (MTX) inhibited tumor growth more than free MTX (with or without heating) at equivalent concentrations. The study also showed that combining the liposomes with heat resulted in a 2.8-day delay in growth compared to 0.7 days when tumors were treated with heat alone. This can be explained by the fact that thermosensitive liposomes are composed of lipids that transition sharply between their fluid and quasi-crystalline states at their phase transition temperature. Near this temperature, liposomal membranes begin to permeate and destabilize, releasing their contents. TSL’s responsiveness to ultrasound thermal effects was demonstrated later by Dromi et al. [78] in 2007, where pulsed high-intensity focused ultrasound (HIFU) was used to induce hyperthermia and trigger Dox release from low temperature-sensitive liposomes (LTSLs). The *in vitro* results showed that 35%-Dox release was achieved at a temperature of 39 °C and a 50% release was observed at 42 °C at the end of a 2 min incubation period. The temperature was then maintained at 42 °C, and the release reached 100% after 12 min. In contrast, no Dox release was detected from Doxil, which served as non-TSLs (NTSLs), even after testing at 42 ℃ using a maximum duration of 12 min. 

Thomas and Lin [79] investigated the effect of several physical factors on liposomes’ responsivity to 20-kHz LFUS. The release of a self-quenching fluorescent dye (calcein), from PEGylated dipalmitoyl phosphatidylcholine (DPPC) liposomes, was studied at different temperatures. It was reported that a power density threshold of 1.5 $\frac{W}{cm^{2}}$ was required to observe significant calcein leakage for all temperature values. Above this threshold, all liposome samples, at the temperatures tested, showed the same upward release trend due to the onset of cavitation, resulting in pore-like defects in the liposome membrane. In general, it was shown that the liposomes’ responsivity to 20-kHz insonation increased as temperature increased, with the highest leakage enhancement observed near the chief lipid’s (DPPC) phase transition temperature (around 41 °C). The work of Magin and Niesman [80] showed that temperature-sensitive LUVs, composed of DPPC and dipalmitoyl phosphatidylglycerol (DPPG), can encapsulate a large amount of the drug and are stable below their transition phase temperature. A complete and rapid drug release happened at the lipid’s phase transition temperature of 43 °C in a mere of seconds. Although LUVs demonstrated some advantages over SLVs in terms of drug loading capacity and release, they are more likely to encounter problems inside the biological system, such as the rapid clearance by the RES cells in the liver and spleen. Despite these limitations, it was hypothesized that the rapid and almost complete drug release in localized hyperthermic regions would result in substantial drug uptake by target cells before the therapeutic dose accumulates in the liver and spleen. However, the latter argument requires more *in vivo* investigations. Although the study above [80] did not involve the use of the ultrasound stimulus, it showed the potential of mild hyperthermia in triggering the drug release. Furthermore, the combined thermal and mechanical effects of ultrasound (hyperthermia and cavitation, respectively) would be expected to further enhance drug leakage from thermosensitive liposomes, compared to hyperthermia alone [81].

Altering the surface charge of thermosensitive liposomes can enhance targeting tumor vasculature. It was shown that positively charged TLSs accumulate preferentially in tumor vessels, thereby potentially inhibiting tumor vascular growth [82,83]. This is attributed to the fact that tumor endothelial cells abundantly express negatively charged membrane proteins [84]. Although cationic TSLs (CTSLs) have shown several advantages, their clinical translation is hampered because of their rapid clearance by the RES and short circulation times. To overcome such drawbacks, PEG molecules can be incorporated into the CTSL’s surface [85]. However, PEGylation of these positively charged particles may obstruct their electrostatic interaction with the negatively charged target cells, reducing the therapeutic efficacy [86]. Additionally, PEGylation can also hinder drug release from liposomes and their endosomal escape after they are internalized by cells [87,88]. Recently, Petrini et al. [89] functionalized CTSLs with a novel synthetic phospholipid 1,2-dipalmitoyl-sn-glycero-3-phosphodiglycerol $\left({\mathrm{DPPG}}_{2}\right)$ to overcome the problems posed by PEGylation, while maintaining a long blood circulation time. Both PEG- and ${\mathrm{DPPG}}_{2}$- modified CTSLs were composed of the same amount of cationic (7.5 mol%) and anionic (5 mol%) lipids and loaded with Dox. Mild hyperthermia (40–43 °C) was combined with the treatment to trigger drug release from the vehicles. Characterizing the synthesized formulations showed a substantial difference in their zeta potentials in saline. On the one hand, ${\mathrm{DPPG}}_{2}$-CTSLs exhibited a detectable positive charge, while, on the other hand, PEG-CTSLs were approximately neutrally charged. This indicates that, unlike ${\mathrm{DPPG}}_{2}$, PEG molecules greatly cover the CTSLs’ positive surface charge, thereby reducing its exposure to the surrounding environment. Indeed, when rat soft tissue sarcoma cells (BN175) and Human umbilical vein endothelial cells (HUVECs) were incubated with the prepared liposomes, ${\mathrm{DPPG}}_{2}$-based CTSLs showed a 3-fold better targetability compared to PEG-based CTSLs. Although ${\mathrm{DPPG}}_{2}$ can enhance liposomal binding and uptake by cancer cells, a minimum of 30 mol% in the lipid bilayer is required to (1) obtain prolonged circulation times and (2) achieve heat-triggered drug release [90,91]. Accordingly, Petrini et al. [89] assessed the effect of ${\mathrm{DPPG}}_{2}$ composition on liposomal thermosensitivity. It was reported that TSLs containing 5 mol% of ${\mathrm{DPPG}}_{2}$ failed to release the encapsulated Dox when heated to 41 °C. In contrast, formulations composed of 30 mol% ${\mathrm{DPPG}}_{2}$ showed a significant Dox release at the same temperature. Furthermore, the inclusion of cationic lipid 1,2-dipalmitoyl-3-trimethylammonium-propane (DPTAP) with either PEG-TSL or ${\mathrm{DPPG}}_{2}$-TSL enhanced their heat sensitivity. 

Cressey et al. [92] formulated image-guided TSLs (iTSLs) that co-deliver two anticancer drugs: (1) SN-38 (in the lipid bilayer) and (2) carboplatin (in the aqueous core). Thermosensitive liposomes must maintain their heat sensitivity after drug encapsulation. Because SN-38 (a hydrophobic drug) resides within the lipid bilayer, it is highly likely to alter the formulated liposome’s phase transition temperature. The synthesized iTSLs were found to be able to accommodate up to 5 wt% of SN-38 while still maintaining a phase transition temperature within the mild hyperthermia region. The other hydrophilic drug (carboplatin) did not alter the thermosensitivity much as it resides inside the liposomal aqueous core. *In vitro* drug release was then assessed by heating the liposomal formulations at varying temperatures (from 25 to 45 °C). While 43.2% of the entrapped carboplatin leaked at 42 °C followed by a complete release at 45 °C, no SN-38 release was detected. This is expected as SN-38 is strongly retained within the lipid bilayer. iTSL disassociation is required to form lipid-based micelles that subsequently can carry the encapsulated hydrophobic SN-38 into cancer cells. Furthermore, the combination of focused ultrasound (FUS) with iTSLs showed increased therapeutic efficiency in treating tumor-bearing mice (injected with MDA-MB-231 cells), compared to iTSLs alone. It was observed that the short FUS treatment (10 min and 42 °C) reduced tumor growth substantially and prolonged survival times. The researchers suggested that the single and short rounds of FUS can help avoid skin damage and off-target drug release that can happen when prolonged heating (pre- and post-injection) is employed, all while enhancing tumor uptake and local cytotoxicity.

To sensitize liposomes to mild hyperthermia, the main lipid, as well as the composition of the lipids within the bilayer membrane, must be selected appropriately. Commonly, DPPC is usually utilized as the main lipid to synthesize thermosensitive liposomes, as it has a main phase transition temperature (around 42 °C) that falls within the mild hyperthermia region. However, to increase the stability of DPPC-based liposomes and prevent pre-leakage of the drug, other lipids with longer acyl chains (higher phase transition temperature) are usually added to the formulations, such as hydrogenated soy phosphatidylcholine (HSPC) and 1,2-disteraroyl-sn-glycero-3-phosphocholine (DSPC) [93]. Recently, Gaál et al. [94] tested the effect of varying DPPC and HSPC compositions on liposomal thermosensitivity. The PEGylated liposomal formulations encapsulated a toxic copper complex as an anticancer agent. Liposomes with different DPPC/HSPC weight ratios were prepared and tested for their *in vitro* anticancer activity against HT-29 human colon adenocarcinoma and C26 mouse colon carcinoma. It was shown that none of the tested formulations presented significant *in vitro* cytotoxicity without heating, indicating their stability and successful drug encapsulation. However, after heat treatment, each formulation demonstrated a distinct release behavior. Liposomes with no or 50 wt% DPPC content failed to release their entrapped cargo at 39 °C. In contrast, those containing 80, 90, and 100 wt% DPPC presented a significant cytotoxic effect upon heating only to 38 ℃. Based on the obtained results, it was determined that a PEGylated liposomal formulation with lipid content of 70 wt% DPPC and 30 wt% HSPC can remain stable at 37 and 38 °C but efficiently release the anticancer agent at 39 °C. 

In another study, Bejarano et al. [95] prepared DPPC-based thermosensitive liposomes that are loaded with the cardioprotective peptide (angiotensin-(1-9)) and modified with gold nanoclusters. Both DSPC and 1-stearoyl-2-hydroxy-sn-glycerol-3-phosphocholine (MSPC) were incorporated into the lipid bilayer to obtain a phase transition temperature a few degrees above the average body temperature. Gold nanoclusters were added to the surface of the liposomes to sensitize them to near-infrared irradiation (NIR) to obtain a localized and controlled temperature-induced release. This is achievable because nanoscale gold particles can efficiently absorb NIR light and convert its energy into heat which can generate hyperthermia. Indeed, the liposomal formulation demonstrated an efficient leakage of the encapsulated cargo upon heating at the phase transition temperature. It was reported that the inclusion of the lysolipid (MSPC) into the lipid membrane enhanced the amount and rate of drug release. This is because when lysolipids are heated at the bilayer’s phase transition temperature, they generate nanopores within the bilayer membrane, through which the drug leaves the vesicle, rather than just enhancing the drug’s bilayer solubility [95,96]. 

Several studies have been conducted to better understand the factors which affect the sonosensitivity of liposomes upon exposure to US as an external triggering modality, aiming to obtain sustainable drug release profiles [97,98,99,100]. As Evjen et al. [101] demonstrated, phospholipid type, as well as its composition within the lipid bilayer, can be altered to increase liposomal sonosensitivity. The study compared US-mediated drug release from two Dox-loaded liposomal formulations, each composed of a different primary lipid: 1,2 distearoyl-sn-glycero-3-phosphatidylethanolamine (DSPE) and DSPC. After 6 min of 40-kHz US irradiation, DSPC-based liposomes released only 9% of the encapsulated Dox. However, when DSPC was replaced by DSPE, this percentage rose sharply to 69%, indicating its high susceptibility to ultrasound. No release was observed from any tested formulations before US exposure, confirming that the detected leakage was due to sonication. Based on these obtained results, DSPE was selected as the main lipid for liposomes, and the effect of its composition within the liposomal membrane on acoustic sensitivity was then evaluated. The increase of DSPE content has been shown to enhance the liposomes’ responsivity to the ultrasound as the optimal formulation yielded a sevenfold increase in the release extent compared to the reference liposomes. 

Besides lipid type and composition, liposomes were also found to be more sensitive to low-frequency ultrasound (LFUS) than high-frequency ultrasound (HFUS), as reported by Cohen-Levi et al. [102]. Dox release from Doxil, induced by LFUS at 20 kHz and HFUS at 1 and 3 MHz, was assessed. It was shown that LFUS released 85% of Dox in saline and 61% in human plasma. When 1-MHz HFUS was applied, these percentages dropped sharply to 58% and 5% in saline and human plasma, respectively. In addition, irradiating US at 1 MHz reduced the release rate compared to 20-kHz LFUS. The use of 3-MHz HFUS showed even lower release levels. The detected increase in drug release at lower frequencies supports the fact that LFUS generates more cavitation events that ultimately lead to more drug leakage from the liposomes in contrast to HFUS [102]. Thus, the application of ultrasound at higher frequencies would be expected to require higher power densities than LFUS to increase the degree of cavitation and enhance liposome disruption [79]. The lower release levels in human plasma may be due to the presence of plasma proteins that absorb a great deal of the ultrasonic energy, thus reducing the extent of cavitation [102]. 

Similarly, Pong et al. [103] reported the same conclusion above in which LFUS is more efficient than HFUS as a liposomal drug release trigger when applied at the same power density. Their work included the synthesis of PEGylated egg phosphatidylcholine vesicles, with sizes ranging from 100 nm to 1 µm. The formulations were loaded with the same leakage assay as proposed by Ellens et al. [104], containing mainly 8-aminonaphthalene-1,3,6-trisulfonic acid, disodium salt (ANTS), and p-xylene-bis-pyridinium bromide (DPX). When encapsulated in the liposomal core, DPX quenched ANTS fluorescence due to the proximity of the molecules. After leakage, DPX and ANTS molecules diffused into the surrounding medium, restoring ANTS fluorescence. This increase in ANTS fluorescence intensity was monitored to evaluate the leakage of the liposomal payload upon US exposure at three different frequencies: 1.6 MHz, 1.0 MHz, and 20 kHz. While 20-kHz ultrasound resulted in a complete release for all liposome sizes tested (100, 300, and 1000 nm), no leakage was detected for sizes less than 100 nm when 1.0-MHz and 1.6-MHz US were used. However, feasible release was recorded when large liposomes (300 and 1000 nm) were irradiated with HFUS. The obtained results suggest that LFUS generates more leakage when compared to HFUS and that the vesicles’ responsivity to US enhances as their diameter increases. The latter can be explained by the Laplace formula: $\frac{\Delta P}{\Delta x}=\frac{2\gamma }{r^{2}}$, describing the pressure gradient $\left(\frac{\Delta P}{\Delta x}\right)$ across a spherical interface, where γ is the interfacial tension, and r is the liposome radius. According to this equation, a tenfold increase in the vesicle radius reduces the pressure gradient required to perturb the lipid bilayer by a factor of one hundred. Therefore, larger liposomes are expected to release more drugs than smaller ones upon sonication [103]. Large liposomes may, however, not be as effective for *in vivo* use since their size may hinder their penetration through the tumor endothelium and render them susceptible to RES clearance. 

The mechanism of US-induced drug release from liposomes is not yet fully understood, which might partly be because of the methodological limitations in observing the reversible changes that occur in the liposome membrane only while ultrasound is applied. Nevertheless, many studies have evaluated the impact of relevant physical and chemical factors on liposome disruption and have proposed some possible mechanisms accordingly. 

Schroeder et al. [105] experimented with measuring the release of three different types of drugs from PEGylated liposomes using ultrasound. All liposomal formulations had the same bilayer lipid composition (hydrogenated soybean phosphatidylcholine as the main lipid) and were the same size (about 100 nm). The three encapsulated drugs were Dox, methylprednisolone hemisuccinate (MPHS), and cisplatin. All three formulations were exposed to 20-kHz LFUS for up to 3 min and demonstrated a maximum drug release of 80% regardless of the encapsulated drug type or its loading method. Instead, it was shown that the amount of the released drug was a function of ultrasound’s amplitude and actual exposure time. The dependence of drug release on LFUS amplitude was shown to be biphasic and linear, with each phase having a different slope. The first phase had a slope of 3.9 $\frac{\%release}{W/cm^{2}}$, whereas that of the second was 16.1 $\frac{\%release}{W/cm^{2}}$. The slope increase occurred at a power density of 1.3 $\frac{W}{cm^{2}}$, and it implied a faster release rate. This behavior can be explained by the onset of trainset cavitation above the specified threshold, which disturbs the liposome membrane to a greater extent, thus increasing the rate and amount of drug release. It was shown that the size of these PC-based liposomes remained the same after ultrasound treatment, indicating that ultrasound did not cause substantial damage to the liposomes’ structure; instead, it introduced pore-like defects in the bilayers through which the drug escaped the vesicles. In addition, the researchers investigated whether the enhanced liposomal permeability is prolonged or confined only to the US irradiation period. In this regard, liposomal suspensions were exposed to 3.3 $\frac{W}{cm^{2}}$ LFUS for 30–180 seconds, and drug release was measured directly after exposure and after 72 h. The percentages were reported to be the same for both times, indicating that once LFUS irradiation was terminated, the defects in the membrane resealed, preventing any further drug leakage. 

**Figure 6:**
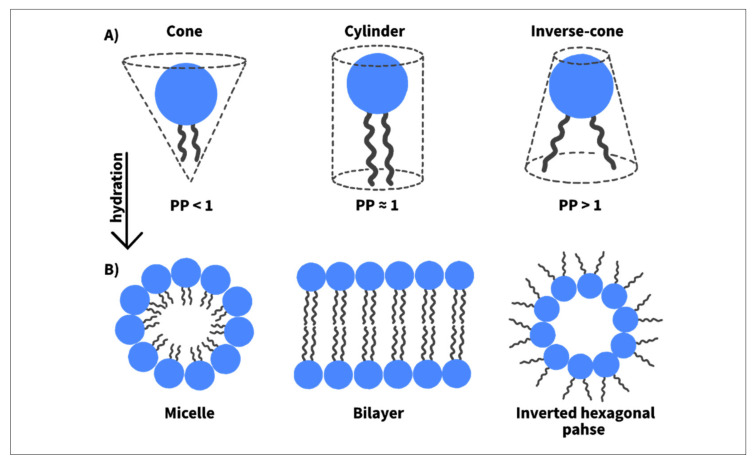
(**A**) Classification of phospholipids based on their geometry and packing parameters; (**B**) phospholipid arrangements when hydrated.

As discussed, while US-induced inertial cavitation triggers drug release, the release profiles are a function of the liposomes’ lipid types and/or compositions [106,107,108,109]. This indicates that the release mechanism is not solely determined by the cavitation events but also depends on the type of phospholipids composing the liposome membrane and how susceptible they are to ultrasound. In general, phospholipids can assume one of three shapes: cone, cylinder, or inverse cone shape. These three geometries differ in their packing parameter (PP), which is defined as the ratio of the cross-sectional area of the apolar (acyl chains) to the polar (headgroup) regions in the phospholipid molecule. For example, conically shaped phospholipids have a PP of less than 1, as they have smaller acyl chains as compared to their headgroups. On the other hand, cylindrical-shaped phospholipids have a PP of 1, as the cross-sectional area of their headgroups is approximately the same as this of their acyl chains. Lastly, inverse-cone phospholipids have a PP above 1, as the cross-sectional area of their acyl chains is larger than this of their headgroups (Figure 6A) [110,111]. 

Because of their dissimilar geometries, phospholipids behave differently when hydrated and aggregate to form layers of different structures. For instance, cylindrical-shaped phospholipids tend to aggregate and form a bilayer by self-assembling into a liquid crystalline lamellar structure ($L_{\propto })$, while inverse-cone phospholipids form a non-bilayer inverted-hexagonal structure ($H_{\mathrm{II}})$ (see Figure 6B) [110,111]. Dioleoyl phosphatidylethanolamine (DOPE), for example, spontaneously assembles to form reversed hexagonal structures when hydrated at temperatures above 10 °C, whereas, below this temperature, a bilayer membrane is formed. The tendency of DOPE to form an $H_{\mathrm{II}}$ phase depends on the saturation degree of its acyl chains and temperature. Such ability of certain phospholipids to transform their membrane geometry from $L_{\propto }$ to $H_{\mathrm{II}}$ can be exploited in drug delivery to initiate pore-like defects in the liposomal membrane through which the drug leaves the liposome. This can be done by the inclusion of appropriate amounts of a non-bilayer forming (conically shaped) phospholipid within the liposome bilayer membrane to introduce local instabilities, as demonstrated by Kang et al. [112]. It was suggested that these instabilities are not enough to perturb the liposomal membrane integrity without applying a stimulus. However, when a trigger is applied, the transformation from lamellar to non-lamellar structure takes place, imparting defects in the membrane and thus allowing for drug release. When the stimulus is terminated, the defects are expected to self-heal, stopping any further leakage. To confirm this hypothesis, two liposome formulations were synthesized: 1,2-dioleoyl-sn-glycero-3- phosphocholine (DOPC)-based liposomes and (DOPE)-based liposomes (containing both DOPC and DOPE). Both formulations were exposed to 20-kHz ultrasound. DOPE-based liposomes demonstrated a higher sonosensitivity than DOPC-based liposomes, indicated by the higher calcein release upon the same exposure time [112]. The former is suggested to be more effective in destabilizing the membranes when sonicated because it tends to form non-bilayer structures, whereas the latter tends to form a stable bilayer [113]. Furthermore, the structural change of these liposomes upon ultrasonic exposure was observed using small angle X-ray scattering. In the absence of ultrasound, DOPE-containing liposomes mostly presented a lamellar phase; however, when they were exposed to ultrasound energy, their membrane underwent a structural transformation through which an inverted hexagonal phase became the dominant phase in the membrane. Conversely, DOPC-based liposomes showed only a lamellar phase before and after ultrasound treatment, indicating that the presence of DOPE molecules in the membrane is the reason behind the transformation to $H_{\mathrm{II}}$ phase and the increased responsivity to ultrasound [112].

However, in contrast to DOPE’s pore-mediated mechanism, Evjen et al. reported a distinct mechanism that involved liposomal destruction upon US exposure, thereby releasing the drug. Specifically, Evjen et al. [97] investigated the kinetic mechanisms of US-mediated drug release from liposomes with two different main lipids: DOPE and hydrogenated soy L-α-phosphatidylcholine (HSPC). The liposome suspensions were analyzed for: (1) size, (2) size distribution, and (3) morphology, before and after US application at 40 kHz. The experiments revealed a significant change in these three parameters for the DOPE-based liposomes after ultrasound treatment, but the HSPC-based liposomes remained intact and maintained their size, with many vesicles not containing Dox in their structure. This suggests that both liposomal formulations interacted differently with ultrasound, resulting in distinct drug release mechanisms. The researchers suggested that sonication led to irreversible disruption of the DOPE-containing liposomes, followed by drug release. Conversely, the unchanged size and structure of the HSPC liposomes indicate a pore-mediated release mechanism. The different US interactions with the lipid bilayers and the corresponding release mechanism can affect the ultrasound’s efficiency on both liposomes and biological tissues. 

**Figure 7:**
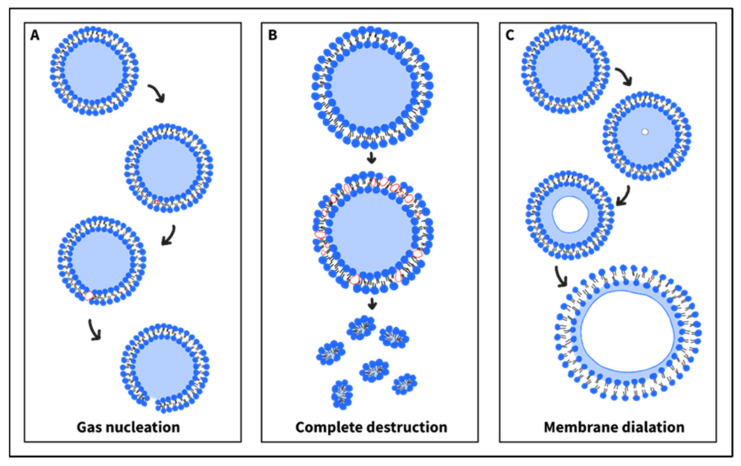
(**A**) Ultrasound might initiate gas nucleation within the lipid bilayer. Subsequent expansion of these gas nuclei during the ultrasound’s negative pressure might result in pore formation in the membrane. (**B**) Multiple poration within the bilayer may lead to its destruction, which might be followed by the formation of micelles. (**C**) The ultrasound rarefaction phase might vaporize dissolved gases or evaporate water in the aqueous core of the liposomes. When expanded, they dilate the bilayer, thus enhancing its overall permeability.

Several mechanisms have been proposed to explain how ultrasound induces sonoporation of liposome membranes and concomitant drug release, even with the absence of cavitation in the surrounding media. Schroder et al. [54] suggested that exposing liposomes to an ultrasonic field may result in the formation of gas bubble nuclei in the hydrophobic region of the lipid bilayer. The subsequent bubble’s expansion (during the US rarefaction phase) or translocation might generate pore-like defects in the membrane providing a path for drug escape into the exterior aqueous surrounding (Figure 7A). When sonication is terminated, the lipid membrane retrieves its initial impermeability. However, the formation of multiple pores in a single liposome membrane may lead to the complete destruction of the formulation. The dissociated phospholipids might then aggregate, forming smaller micellar vesicles (lipid-based monolayered vesicles) (Figure 7B). Similarly, Wrenn et al. [114] have hypothesized that some gas molecules might partition from the aqueous surrounding into the lipid bilayer, leading to the nucleation of bubbles within the membrane. These nanobubbles can then expand and contract upon low-frequency ultrasound application, leading to local disruption in the liposomal membrane, followed by drug leakage. A mathematical model was derived, predicting that the bubble’s nucleation possibility increases as the bilayer’s thickness decreases. The latter can be achieved by the incorporation of cholesterol into the membrane, as it is known to affect the packing and, therefore, the thickness of the bilayers. Another mechanism was proposed that did not suggest the formation of local pore-like defects. Instead, it speculated an overall enhanced membrane’s permeability upon sonication [115]. In this hypothesis, ultrasound may trigger nucleation of dissolved gases (e.g., oxygen, carbon dioxide, or nitrogen) or vaporization of water molecules, inside the aqueous core. The subsequent expansion of the gas or vapor dilates the liposome membrane by increasing the separation between the phospholipid molecules (Figure 7C). This enhanced permeability will then allow for drug leakage [115].

Based on the studies discussed, drug release occurs due to mechanical disruptions that ultimately destabilize the liposomal membrane, rather than because the liposomes are inherently sensitive to ultrasound. This insensitivity is due to the absence of gas bubbles in the liposome structure that can instigate cavitation events inside the formulation. Hence, the inclusion of gas bubbles or nanoemulsions in liposomes to act as cavitation nuclei may render them susceptible to US. Liposomes of this type are called echogenic or bubble liposomes, and they have been studied recently [116,117,118]. Lattin et al. [119] formulated eLiposomes (liposomes containing liquid emulsion droplets), using either perfluoropentane $\left({\mathrm{PFC}}_{5}\right)$ or perfluorohexane $\left({\mathrm{PFC}}_{6}\right)$ as emulsions. A release mechanism is proposed in which the local pressure drops below the vapor pressure of the emulsion, during the US rarefaction state. Consequently, the liquid nanodroplets convert into nanobubbles which then expand inside the liposomes to eventually break them open. Calcein release from eLiposomes, upon 20-kHz US exposure, was tested. Different emulsion sizes were studied: 100 and 400 nm. The results showed that eLiposomes increased calcein release by 3 to 5 times compared to control liposomes. Furthermore, 400-nm emulsions promoted more liposomal drug release than 100-nm emulsions. However, liposomes with large emulsions may not benefit from the tumor EPR features and thus may fail to accumulate at target sites. Liposomes containing small emulsions will be more suitable in a biological system. The *in vitro* results also showed that the ${\mathrm{PFC}}_{5}$ droplets were more sensitive to US than ${\mathrm{PFC}}_{6}$, as their liposomes exhibited higher levels of calcein release. Consistent with other studies, the extent of release was reported to depend on the ultrasonic intensity and exposure time. 

The studies discussed above demonstrated the ultrasound’s ability to trigger drug release from liposomes, explored the influence of different factors on the liposomes’ acoustic sensitivity, and proposed some release mechanisms. The following studies, however, involve some cellular *in vitro* and *in vivo* experiments that have been conducted to test the therapeutic efficacy of liposomal systems with ultrasound as a trigger in cancer treatment.

In the same work described earlier [105], Schroeder et al. assessed the potency of LFUS-released cisplatin by measuring its cytotoxicity against cisplatin-sensitive C26 murine colon adenocarcinoma cells. Stealth liposomal cisplatin was added to the cell cultures, followed by US irradiation. Higher levels of cisplatin release were reported with increasing US exposure time. Compared to unirradiated free cisplatin, equal amounts of LFUS-released cisplatin exhibited similar cytotoxicity in the treated cells. The latter finding indicates that LFUS altered neither cisplatin’s chemical integrity nor its biological activity.

Modification of liposome surfaces with targeting ligands promotes higher rates of cellular internalization and thus enhanced cytotoxicity, compared to control liposomes or free drugs [120,121,122]. A study by Elamir et al. [123] showed that decorating liposomal membranes with Trastuzumab (TRA), an anti-HER2 monoclonal antibody, increased the liposomes’ cytotoxicity and their cellular uptake by HER2-positive breast cancer cells, compared to control liposomes. For this, two cell lines were used: HER2 + (SKBR3) and HER2- (MDA-MB-231), and incubated with either control or TRA-modified liposomes for 4 h. Cytometry analysis was then performed to measure calcein fluorescence intensity within SKRB3 cells. Fluorescence was reported to be stronger in cells treated with TRA liposomes (25,160 a.u.), compared to those treated with controls (7236 a.u.), suggesting that TRA ligands led to more calcein uptake. The fluorescence intensity in the cells incubated with TRA-liposomes was further enhanced to 32,735 a.u. when exposed to 35-kHz LFUS for 5 min, and it was more than that achieved by control liposomes alone by a factor of 4.5. It was suggested that ultrasound enhanced the permeability of liposomes and cell membranes through sonoporation, resulting in a better calcein release and uptake. Furthermore, the increase in calcein fluorescence intensity by TRA-conjugated liposomes over the controls may be due to the binding of TRA ligands to HER2 receptors overexpressed by SKRB3 cells, leading to increased cellular uptake levels. The same test was repeated to verify this hypothesis with MDA-MB-231 cells (with low expression of HER2 receptors). Both control and TRA liposomes achieved approximately similar fluorescence intensities of 10,022 and 13,914 a.u., respectively, in the tested cells. This was explained by the low expression levels of HER2 receptors on MDA-MB-231 cells, which reduced the targeting specificity of TRA-conjugated liposomes, lowering the extent of cellular internalization compared to SKBR3 cells. This work demonstrated that the combination of TRA-targeted liposomes with LFUS is a non-invasive and effective breast cancer therapeutic tool that can be further investigated in future *in vivo* and clinical work to evaluate its full therapeutic potential.

AlSawaftah et al. [124] assessed the targeting specificity of calcein-loaded transferrin (Tf)-conjugated liposomes to HeLa cells by analyzing calcein cellular uptake, compared to non-targeted (control) liposomes. HeLa cells incubated with transferrin-modified liposomes showed significantly higher calcein uptake than control liposomes. The overexpression of Tf receptors on Hela cells induced receptor-mediated endocytosis upon binding to Tf, triggering greater uptake of the liposomal dye. Furthermore, HeLa cells, incubated with control liposomes, were sonicated with 35-kHz LFUS, enhancing calcein uptake by 18% compared to the non-sonicated cell system treated with control liposomes. Furthermore, HeLa cells showed greater calcein uptake when incubated with Tf liposomes and irradiated with LFUS, with an increase of approximately 42% over the unirradiated cell culture medium. Combining active targeting with LFUS demonstrated a 151% enhancement in calcein uptake by HeLa cells compared to non-sonicated controls. These findings support the work by Elamir et al., discussed earlier [123], emphasizing the synergetic effect of ultrasound and targeted liposomes to improve the release and uptake of the encapsulated therapeutic payloads. However, extensive research is needed to ensure that liposomes are stable *in vivo* and that the drug retains its therapeutic activity after ligand attachment.

Initially, microbubbles were developed as contrast agents in diagnostic ultrasound. In recent years, their use as both delivery vehicles and cavitation nuclei, to improve gene and drug delivery, has been investigated [125,126]. These gas-filled cavities range in diameter from 0.5 to 1 µm and are coated with an outer shell to prevent them from dissolving, thereby stabilizing them in biological systems. The microbubble’s shell can be formulated using lipids or polymers, and the drug can be entrapped either within the shell or attached directly to its surface. Under ultrasound exposure, these microbubbles begin to cavitate and collapse, liberating the encapsulated drug into the tumor vicinity [127]. 

Despite their potential to enhance drug and gene therapy, microbubbles exhibit some limitations when used *in vivo*. Due to their large size, they cannot easily extravasate through the defective tumor vasculature. Even after extravasation, the high tumor interstitial pressure, dense extracellular matrix, and tight intercellular spaces with a mean diameter of 1.7 µm limit the microbubble diffusion rates through the tumor interstitium, thereby lowering the drug concentration at the desired regions. In addition, these formulations possess low blood stability, as they are rapidly cleared by the RES, which ultimately reduces their lifespan in the body and their concentration at the tumor site [25,127].

Currently, some studies have been aiming at combining the advantages of liposomes and microbubbles for better ultrasonic therapeutic activity [128,129]. Lentacker et al. [130] conjugated biotinylated Dox-containing liposomes to the shell of a biotinylated microbubble via avidin molecules and evaluated this system’s cytotoxicity against melanoma BLM cells, upon US irradiation. Exposure of cells to liposomal-Dox-loaded microbubbles and 1-MHz US resulted in a two-fold increase in cell death compared to exposure to liposomal Dox and ultrasound. When cells were incubated with the liposome-microbubble system, instantaneous Dox uptake by the cells was observed, with most of the released Dox detected in the nuclei of cancer cells. On the contrary, when cells were treated with Dox-loaded liposomes, the majority of the released drug was present in the cells’ cytoplasm. It was speculated that the faster and higher Dox uptake, associated with the microbubble formulations, is due to the high extent of sonoporation induced by the synergistic effect of ultrasound and microbubbles. US exposure resulted in cavitation and implosion of the microbubbles, followed by perforation of liposomes and cell membranes, leading to greater drug release and uptake, respectively, compared to Dox-loaded liposomes. Based on the obtained *in vitro* results, the research group believes that this liposomal-Dox-microbubble system could be a promising tool to enhance the US-triggered Dox delivery *in vivo*, as both liposomal Dox (Doxil) and microbubbles are already used in the clinic. While such an approach could successfully enhance the drug’s loading capacity and release [130], the micron-sized liposome-bubble system will not benefit from the tumor EPR effect and extravasate readily into the tumor interstitium due to their relatively large size [131]. Nevertheless, the local application of ultrasound at the tumor site, besides liberating the drug, might also increase the permeability of tumor endothelium, as suggested by Yuh et al. [132], allowing extravasation of the microbubble system. To confirm this possibility, *in vivo* experiments should be performed by which the effectiveness of such a system can be assessed. 

**Figure 8:**
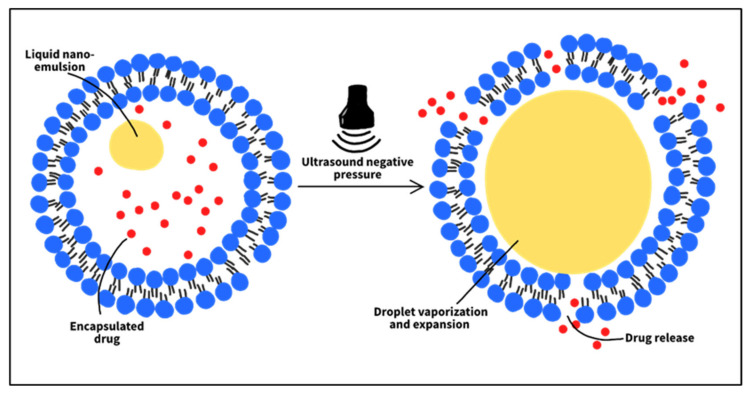
Liposomes containing nano-emulsions (emulsion liposomes). When exposed to ultrasound’s negative pressure, the nanodroplet vaporizes and expands inside the aqueous core, disturbing the liposomal membrane and leading to drug release.

An alternative to pre-existing microbubbles is the development of liposome-nanobubble systems or emulsion liposomes that utilize the phenomenon of acoustic droplet vaporization (ADV) to induce drug release (see Figure 8). Prabhakar and Banerjee [133] conjugated paclitaxel-loaded liposomes to the surface of a nanobubble (NB-PTXLp) to form a complex with a mean size of 528.7 nm. The cytotoxicity of the developed formulation combined with 1-MHz ultrasound was tested against four cell lines: MiaPaCa-2, Panc-1, MDA-MB-231, and AW-8507. It was shown that NB-PTXLp exhibited higher cytotoxicity by several folds in all cell lines tested compared to the commercial paclitaxel-containing formulation ABRAXANE (nanoparticle albumin-bound paclitaxel). In this case, the ultrasound burst the nanobubble in the NB-PTXLp complex, which resulted in the sonoporation of liposomes and cancerous cells, thus allowing the drug to enter target cells more effectively. Despite the promising *in vitro* results, preclinical tests should be performed to evaluate NB-PTXLp efficiency before clinical usage.

The work of Javadi et al. [134] exploited the ADV phenomenon to liberate calcein from folated eLiposomes. ${\mathrm{PFC}}_{5}$ emulsions were successfully encapsulated in calcein-loaded folated liposomes. The effect of folate ligand and ${\mathrm{PFC}}_{5}$ emulsion in delivering calcein into HeLa cells under ultrasound irradiation was evaluated. A measurable calcein release required the combination of emulsions and sonication, as a minimal release was obtained when used separately. The study also revealed that folated eLiposomes delivered significant calcein to HeLa cells, compared to non-folated eLiposomes or when folate receptors on HeLa cells were blocked, where in the latter two cases, only a negligible calcein uptake was detected. It was speculated that this low intracellular delivery is because a large number of liposomes were not endocytosed by cancer cells. The proposed formulation of this study offers a triple-targeted drug delivery system: (1) Its small size permits the passive extravasation through the defected tumor endothelium, (2) folate ligands incorporated onto the liposome’s surface can actively target folate receptors on cancer cells, inducing receptor-mediated endocytosis, and lastly, (3) local application of ultrasound triggers the droplet vaporization, thus rupturing the liposomes at the tumor site and allowing for spatiotemporal control of drug release. 

Although DOXIL, the first liposomal formulation approved by the FDA, presents many advantages over free DOX, such as longer blood circulation and fewer side effects, its sufficient stable membrane hinders the release of the chemotherapeutic agents from its structure to the tumor vicinity. Therefore, the effect of the anticancer drug is limited by its low accumulation at the tumor site. Thus, to enhance the local release of the chemotherapeutic agents, different stimuli have been combined with DOXIL and tested for their efficiency. One of these stimuli that have been intensively studied recently is ultrasound due to its ease of control and non-invasiveness [135].

Yuh et al. [132] tested Dox release from Doxil^®^ using pulsed 1.5-MHz HIFU in C3H/Km mice bearing a murine squamous carcinoma (SCC7 cell line). The mean Dox concentration in tumors treated with Dox alone was 4.2 µg/g ±0.95, while this measured in tumors exposed to Dox and HIFU was 9.4 µg/g ± 2.1, representing a 124% enhancement. Dextran-fluorescein isothiocyanate was also administered to the mice to observe its extravasation into the tumor vicinity using *in vivo* confocal microscopy. It was shown that, in the control tumors (not exposed to HIFU), the extravasation of the fluorophore conjugate was restricted to the tumor vasculature. In contrast, HIFU application increased the tumor vascular permeability, which was evident in the presence of larger amounts of Dextran-fluorescein isothiocyanate in the tumor interstitium. The authors suggested that cavitation and/or hyperthermia may be responsible for this US-induced release. Another work by Dromi et al. [78] investigated the role of HIFU-induced hyperthermia as a Doxil release trigger in BALB/c mice bearing a murine mammary adenocarcinoma (JC cell line). In contrast to the previous study, the combination of Doxil and pulsed HIFU (used to raise the temperature to 42 °C) did not show significant Dox release compared to Doxil or LTSL alone. However, exposing tumors, injected with LTSLs, to HIFU led to a 3- to 4-fold increase in Dox release in comparison to non-exposed controls, indicating the role of hyperthermia and thermosensitive liposomes in enhancing the local drug delivery in tumors. The lack of Dox release from Doxil at elevated temperatures might be due to its membrane’s high cholesterol content (40% mole fraction). High cholesterol levels introduce a liquid-ordered phase in the lipid bilayer, lowering the membrane’s permeability and temperature sensitivity [136,137]. 

Typically, the PEGylated liposomal formulation, Doxil, remains intact when circulating in plasma, but begins to release its content upon reaching tumor tissues, even when no external stimulus is applied. This suggests that Dox release from Doxil might be due to differences in the microenvironment between healthy and cancerous tissues, which can act as an internal trigger [138]. Although the release mechanism of Doxil has not been clearly defined, Lisa et al. demonstrated that the high ammonia levels at the tumor site, produced by glutaminolysis, enhance the release and, thus, the therapeutic efficiency of the encapsulated Dox [138]. However, other liposomal systems, such as liposomal cisplatin, showed very low release kinetics in the tumor vicinity, leading to poor therapeutic activity [139,140]. To achieve higher release levels, Schroeder et al. [136] used 20-kHz LFUS to study the release of cisplatin from liposomes *in vivo* and to assess its impact on therapeutic efficacy. Mice bearing J6456 murine lymphoma tumors were injected intraperitoneally (i.p.) with cisplatin-loaded liposomes. When the abdominal wall was exposed to LFUS for 2 min, about 70% of the drug was released, whereas less than 3% leaked from the nonirradiated liposomes. To estimate the therapeutic efficiency, BALB/c mice with C26 colon adenocarcinoma tumors were injected intravenously (i.v.) with either liposomal cisplatin or free cisplatin. Tumors were exposed to LFUS after the passage of 24 h upon injection, allowing liposomes to extravasate and accumulate at the tumor site. The results showed that the US-liposome system showed the best therapeutic activity compared to all other treatments, including liposomal cisplatin, free cisplatin with or without LFUS, and LFUS alone. This study showed that the combination of liposomal cisplatin and ultrasound prevented tumor growth and resulted in its regression. 

Pitt et al. [141] investigated the delivery of Dox-loaded PC-based liposomes to bilateral intradermal DHD/K12 tumors implanted in BDIX rats’ hind legs. After injecting the encapsulated Dox into the tail vein, low-frequency ultrasound (20 kHz) was applied to one of the tumors for 15 min. The therapy was repeated weekly for four weeks. The experiment showed that, unlike the non-sonicated tumors, sonicated tumors regressed to unmeasurable sizes, highlighting the potential of ultrasound for site-specific therapy of solid tumors [141]. Furthermore, Evjen et al. [142] explored the *in vivo* effects of ultrasound as a trigger for liposomal drug release. In their work, DOPE-based liposomes were loaded with a near-infrared fluorochrome (Al (III) Phthalocyanine Chloride Tetrasulphonic acid (AlPcS4)) and administered into Balb/c mice inoculated with 22Rv1 human prostate tumor cells. Subsequent exposure of tumors to 1.1-MHz US significantly increased the fluorescence, indicating that ultrasound enhanced the release from DOPE-based liposomes. However, no increase in fluorescence was detected in tumors treated with HSPC-based liposomes and ultrasound, implying that DOPE lipids were more responsive to ultrasound. Table 3, Table 4 and Table 5 summarize the studies discussed in this section.

## 6. Conclusions and Future Prospects

Research has been focused on developing new technologies to minimize the systemic side effects of conventional chemotherapy. As entailed in this paper, nanoparticles hold the key to this endeavor. They can target tumors by three mechanisms: passive, active, and triggered targeting. 

Passive targeting utilizes the leaky nature of the cancer vasculature to deliver a sufficient amount of the drug to the tumor interstitium while preventing its accumulation in healthy tissues. However, due to the wide variety of cancer types that show differences in their microenvironments, passive diffusion cannot always be guaranteed. As for active targeting, ligand–receptor interactions facilitate cellular liposome internalization, increasing the drug concentration in cancer cells. Research shows that this targeting mechanism has a promising future in clinical practice. However, more studies are needed to ensure the stability of the liposomal structure, its drug loading capacity, and its release kinetics after ligand attachment. Furthermore, the therapeutic activity of the drug should not be altered by the active ligands. 

After endocytosis, drug release from the liposomes into the cytosol can be achieved using an internal or external trigger. The liposomes should have the ability to remain intact until a trigger is applied at the tumor site. This character is essential because a leaky liposome will release its payload slowly into the cancer cell without achieving an effective therapeutic concentration, while a stimulus-mediated release will ensure an instant surge in the intracellular drug concentration to reach the lethal level. Although such stimuli may enhance the release profile, an appropriate therapeutic window should be specified within which the desired therapeutic effect is achieved with minimum trigger-related side effects. One of the most researched triggers is ultrasound. The different parameters of ultrasound must be optimized to release the drug from liposomes in the tumor without damaging healthy neighbor cells. 

Ultrasound induces liposomal drug release by giving rise to either mechanical effects, thermal effects, or both. To enhance liposomes’ acoustic sensitivity, researchers have been exploring the inclusion of nano-emulsions or nanobubbles within the liposomal structure. As such, ultrasonic energy will evaporate the nano-emulsion or cavitate the nanobubble, thus disturbing the lipid bilayer and releasing the encapsulated drug. 

In conclusion, both liposomes and ultrasound are being used separately in clinics. Current *in vitro* and *in vivo* studies have demonstrated that combining these two modalities results in a synergistic effect that can potentially improve the therapeutic efficacy and minimize the side effects of traditional chemotherapy. 

## Figures and Tables

**Table 1:** Clinically used liposomal systems for cancer therapy [28,29,30,31,32].

Product	Approval Year	Drug	Lipid Composition (Molar Ratio)	Marketed by	Indication
Doxil^®^	1995	Doxorubicin	HSPC: Cholesterol: ${\mathrm{PEG}}_{2000}$-DSPE (56:39:5)	Sequus Pharmaceuticals	AIDS-related Kaposi’s sarcoma, ovarian cancer, breast cancer
DaunoXome^®^	1996	Daunorubicin	DSPC:Cholesterol (2:1)	NeXstar Pharmaceuticals	AIDS-related Kaposi’s sarcoma
Depocyt^®^	1999	Cytarabine	DOPC, DPPG, cholesterol and triolein, (DepoFoam™)	SkyPharma Inc.	Neoplastic meningitis
Myocet^®^	2000	Doxorubicin	EPC:Cholesterol (55:45)	Elan Pharmaceuticals	Used with cyclophosphamide to treat metastatic breast cancer
Mepact^®^	2004	Mefamurtide	DOPS: POPC (3:7)	Takeda Pharmaceutical Limited	High-grade non-metastatic osteosarcoma
Lipusu^®^	2006	Paclitaxel	PC, cholesterol	Sike Pharmaceutical Co. Ltd.	Gastric, ovarian, and lung cancer
Marqibo^®^	2012	Vincristine	SM: Cholesterol (60:40)	Talon Therapeutics, Inc.	Acute lymphoblastic leukemia
Lipodox^®^	2012	Doxorubicin	DSPC: Cholesterol: ${\mathrm{PEG}}_{2000}$-DSPE (56:39:5)	Sun Pharma	Breast and ovarian cancer
Onivyde™	2015	Irinotecan	DSPC:${\mathrm{MPEG}}_{2000}$:DSPE (3:2:0.015)	Merrimack Pharmaceuticals Inc.	Metastatic adenocarcinoma of the pancreas
CPX-351 (Vyxeos^®^)	2017	Cytarabine: Daunorubicin (5:1)	DSPC: DSPG: Cholesterol (7:2:1)	Jazz Pharmaceuticals	Acute myeloid leukemia

HSPC (hydrogenated soy phosphatidylcholine); PEG (polyethylene glycol); DSPE (distearoyl-sn-glyc ero-phos phoethanolamine); DSPC (distearoylphosphatidylcholine); DOPC (dioleoylphosphatidylcholine); DPPG (dipalmitoylphosphatidylglycerol); EPC (egg phosphatidylcholine); DOPS (di oleoylphosphatidylserine); POPC (palmitoyloleoylphosphatidylcholine); PC (phosphatidylcholine); SM (sphingomyelin); MPEG (methoxy polyethylene glycol); DSPG (distearoylphosphatidyl glycerol).

**Table 2:** Comparison between passive and active targeting.

Passive Targeting	Active Targeting
Alters the drug biodistribution in the body	Increases the cellular drug uptake by target cells
Targeting is based on the Enhanced Permeability and Retention (EPR) effect in tumors	Targeting is based on molecular interactions between NP’s ligands and cancer receptors.
Modest specificity and limited efficacy	High specificity and efficacy
Restricted in use	Very versatile
More side effects and toxicity	Fewer side effects and toxicity
Less effort to engineer and synthesize	More challenging to synthesize NPs with ligands attached, while maintaining their ability to bind with target receptors, to achieve the activity of interest (active targeting)

**Table 3:** *In vitro* release experiments of nanodrugs from liposomes using ultrasound.

Liposome Type	Payload	US Parameters	Overview	References
Low-temperature sensitive liposomes (LTSLs) Non-thermosensitive liposomes (NTSLs)	Doxorubicin	$\mathrm{Pulsed}\;\mathrm{high}-\mathrm{intensity}\;\mathrm{focused}\;\mathrm{ultrasound},\;1300\frac{W}{cm^{2}}$, 10% duty cycle (100 ms ON/ 900 ms OFF)	For LTSLs: ultrasound-induced hyperthermia achieved 35% release at 39 °C and 50% at 42 °C, within 2 min. A complete release was achieved after 12 min of high-intensity ultrasound exposure at 42 °C. For NTSLs: No release was detected.	[78]
PEGylated DSPE PEGylated DSPC	Doxorubicin	40 kHz, continuous mode (100% duty cycle), 20% amplitude	After 6-min ultrasound exposure, DSPE-based liposomes released 69% of the encapsu lated doxorubicin compared to 9% from DSPC-based liposome DSPE lipids enhanced the liposomes’ sonosensitivity, with the optimized formulation resulting in a 7-fold increase in doxorubicin release compared to reference liposomes.	[101]
Doxil	Doxorubicin	20 kHz, 1 and 3 MHz	20-kHz low-frequency ultrasound released more doxorubicin compared to 1- and 3-MHz high-frequency ultrasound.	[102]
PEGylated, egg PC	A buffer composed of p-xylene-bis-pyridinium bromide (DPX) and 8-aminonaphthalene-1,3,6-trisulfonic acid, disodium salt (ANTS).	$20\;\mathrm{kHz},\;0.13\;\frac{W}{cm^{2}}$, 100% duty cycle $1\;\mathrm{MHz},\;3\;W/cm^{2}$, 40% duty cycle $1.6\;\mathrm{MHz},\;46.9\;\frac{W}{cm^{2}}$, 40% duty cycle	Low-frequency ultrasound achieved higher leakage than high-frequency ultrasound. Low-frequency ultrasound induced buffer release for all lipo somal formulations tested. High-frequency ultrasound induced drug release from 300- and 1000-nm vesicles, but not from 100-nm vesicles. Vesicles’ responsivity to ultrasound enhances as their diameter increases.	[103]
PEGylated DPPC	Calcein	$20\;\mathrm{kHz},\;25\%\;\mathrm{duty}\;\mathrm{cycle},\;1\;\frac{W}{cm^{2}}$	Hyperthermia enhanced drug release, with the largest enhancement at the lipid transition temperature (41 °C).	[79]
PEGylated, hydrogenated soybean PC	Doxorubicin Methylprednisolone hemisuccinate Cisplatin	$20\;\mathrm{kHz},\;\mathrm{full}\;\mathrm{duty}\;\mathrm{cycle},\;\mathrm{varying}\;\mathrm{intensities}:\;\mathrm{from}\;0\;\mathrm{to}\;7\;\frac{W}{cm^{2}}$	A maximum release of 80% was reported after 3-min ultrasound exposure for all formulations. $\mathrm{The}\;\mathrm{drug}\;\mathrm{release}\;\mathrm{rate}\;\mathrm{increased}\;\mathrm{substantially}\;\mathrm{after}\;a\;\mathrm{power}\;\mathrm{density}\;\mathrm{threshold}\;\mathrm{of}\;1.3\;\frac{W}{cm^{2}}$. The proposed release mechanism is that the onset of cavitation introduced pore-like de fects in the liposomal membrane, allowing for drug release.	[105]
DOPC DOPE	Calcein	20 kHz, 10-min exposure time, 20% amplitude	DOPE-based liposomes underwent a structural change from lamellar to non-lamellar phase upon sonication, resulting in more drug release, compared to DOPC-based liposomes, which showed a lamellar phase before and after US irradiation.	[112]
PEGylated DOPE PEGylated HSPC	Doxorubicin	$40\;\mathrm{kHz},\;12\;cm^{2}$, continuous mode (100% duty cycle)	Ultrasound interacted differently with different phospholipids leading to distinct drug re lease mechanisms. Suggested mechanisms: – DOPE-based liposomes: irreversible damage and deformed structure that led to drug release. – HSPC- based liposomes: introduction of pore-like defects in the membrane through which drug escaped.	[97]
eLiposomes	Calcein	$20\;\mathrm{kHz},\;\mathrm{intensity}\;\mathrm{was}\;\mathrm{varied}\;\mathrm{between}\;0.5\;\mathrm{and}\;5\;\frac{W}{cm^{2}}$	$\mathrm{Eliposomes},\;\mathrm{containing}\;\mathrm{perfluorohexane}\;\left({\mathrm{PFC}}_{6}\right)$$\mathrm{and}\;\mathrm{perfluoropentane}\;\left({\mathrm{PFC}}_{5}\right)$ nanoemulsions, released 3–5 times more calcein than control liposomes. eLiposomes, with 400-nm emulsions, resulted in a higher calcein release than those containing 100-nm emulsions, upon US exposure. The drug release extent depended on ultrasound’s power density and exposure time.	[119]

DSPE (1,2 distearoyl-sn-glycero-3-phosphatidylethanolamine); DSPC, (1,2 distearoyl-sn-glycero-3-phosphatidylcholine); PC (phosphatidylcholine); DPPC (dipalmitoyl phosphatidylcholine); DOPC (1,2-dioleoyl-sn-glycero-3- phosphocholine), DOPE (dioleoyl phosphatidylethanolamine); HSPC (hydrogenated soy L-α-phosphatidylcholine).

**Table 4:** *In vitro* cell studies using liposomal drugs and ultrasound.

Liposome Type	Payload	Cancer Cell Line	US Parameters	Overview	References
PEGylated	Cisplatin	C26 murine colon adenocarcinoma	$20\;\mathrm{kHz},\;\mathrm{full}\;\mathrm{duty}\;\mathrm{cycle},\;\mathrm{varying}\;\mathrm{intensities}:\;\mathrm{from}\;0\;\mathrm{to}\;7\;\frac{W}{cm^{2}}$	Longer ultrasound exposure time increased the level of cisplatin release. Cytotoxicity of cisplatin released by low-frequency ultrasound was the same as unirradiated free cisplatin. Exposure to ultrasound did not modify the chemical properties or the biological potency of cisplatin.	[105]
PEGylated DPPC, cholesterol with trastuzumab modification	Calcein	HER2 + (SKBR3) HER2- (MDA-MB-231)	$35\;\mathrm{kHz},\;20\;\frac{nW}{cm^{2}}$	SKBR3 cells incubated with trastuzumab-modified liposomes showed higher calcein fluorescence intensity than those incubated with controls. Irradiating the cells (treated with trastuzumab-modified liposomes) with low-frequency ultrasound led to fluorescence intensity enhancement by a factor of 4.5 compared to treating with control liposomes alone. No significant difference in fluorescence intensities inside MDA-MB-231 cells was recorded when incubated with either targeted or control liposomes. The results suggest that trastuzumab ligands targeted the overexpressed HER2 receptors on SKBR3, hence inducing faster calcein cellular internalization.	[123]
PEGylated DPPC, cholesterol with transferrin modification	Calcein	HeLa cells	$35\;\mathrm{kHz},\;1\;\frac{W}{cm^{2}}$	Transferrin-modified liposomes resulted in higher calcein uptake by HeLa cells compared to control liposomes. Low-frequency ultrasound enhanced HeLa cells’ calcein uptake from control liposomes by 18% and from transferrin-targeted liposomes by 42%. Calcein uptake from sonicated transferrin-targeted liposomes was approximately two-and-a-half times higher than that from non-sonicated control liposomes.	[124]
A microbubble modified with PEGylated-DPPC liposomes	Doxorubicin	Melanoma BLM cells	$1\;\mathrm{MHz},\;50\%\;\mathrm{duty}\;\mathrm{cycle},\;2\;\frac{W}{cm^{2}}$	Microbubbles decorated with doxorubicin-loaded liposomes showed a two-fold increase in cell death com pared to doxorubicin-loaded liposomes. Most of the released doxorubicin was detected in the nuclei of cancer cells when incubated with the liposome-microbubble system. Most of the released doxorubicin was present in the cytoplasm of cancer cells when incubated with doxorubicin-loaded liposomes.	[130]
A nanobubble modified with paclitaxel-loaded liposomes (NB-PTXLp)	Paclitaxel	MiaPaCa-2, Panc-1, MDA-MB-231, and AW-8507	$1\;\mathrm{MHz},\;1\;\frac{W}{cm^{2}}$, 75% duty cycle	NB-PTXLp achieved higher intracellular uptake by several folds compared to ABRAXANE.	[133]
Folated eLip osomes	Calcein	HeLa cells	$20\;\mathrm{kHz},\;1\;\frac{W}{cm^{2}}$	$\mathrm{Perfluoropentane}\;\left({\mathrm{PFC}}_{5}\right)$ emulsion and ultrasound combined resulted in measurable release compared to when used separately. Folated eLiposomes enhanced calcein uptake compared to non-folated eLiposomes.	[134]

DPPC (dipalmitoyl phosphatidylcholine).

**Table 5:** *In vivo* studies using liposomal drugs and ultrasound.

Liposome Type	Payload	Cancer Cell Line	US Parameters	Overview	References
Doxil^®^	Doxorubicin	Murine squamous carcinoma (SCC7 cell line), C3H/Km mice	1.5-MHz high-intensity focused ultrasound, Pulsed	Treating tumors with Doxil and ultrasound enhanced doxorubicin accumulation at the tumor site by 124% compared to treating with doxorubicin alone.	[132]
Doxil as non-thermosensitive liposomes (NTSLs) Low-temperature sensitive liposomes (LTSLs)	Doxorubicin	Murine mammary adenocarcinoma (JC cell line), BALB/c mice	$\mathrm{Pulsed}\;\mathrm{high}-\mathrm{intensity}\;\mathrm{focused}\;\mathrm{ultrasound},\;1300\;\frac{W}{cm^{2}}$, 10% duty cycle (100 ms ON/ 900 ms OFF)	The combination of LTSLs and high-intensity focused ultrasound demonstrated a 3- to 4-fold increase in doxorubicin release compared to non-exposed controls. Exposing Doxil with high-intensity focused ultrasound showed no significant increase in release compared to non-sonicated Doxil or LTSLs.	[78]
PEGylated, HSPC	Cisplatin	J6456 murine lymphoma, mice C26 colon adenocarcinoma, BALB/c mice	$20\;\mathrm{kHz},\;5.9\;\frac{W}{cm^{2}}\;$	Release test: Sonicated liposomal cisplatin released 70% of their content compared to only 3% release from non-sonicated liposomes. Therapeutic efficiency: Liposomal cisplatin with ultrasound showed the best therapeutic activity compared to other treatments.	[136]
$\mathrm{PC},\;\mathrm{cholesterol},\;\mathrm{DSPE}-{\mathrm{PEG}}_{2000}$, alpha-tocopherol	Doxorubicin	Colorectal tumor (DHD/K12/TRb cells), BDIX rats	$20\;\mathrm{kHz},\;1\;\frac{W}{cm^{2}}$, continu ous for 15 min	Tumors, treated with low-frequency ultrasound and liposomal doxorubicin, regressed in size significantly compared to those treated with liposomal doxorubicin only.	[141]
DOPE HSPC	Aluminum (III) Phthalocyanine Chloride Tetrasulphonic acid (AlPcS4)	22Rv1 human prostate tumor cells, Balb/c mice	$1.1\;\mathrm{MHz},\;10\;\frac{kW}{cm^{2}}$	DOPE-based liposomes and ultrasound increased the fluorescence in tumors. No increase in fluorescence in tumors treated with HSPC-based liposomes and ultrasound.	[142]

HSPC (Hydrogenated soybean phosphatidylcholine); PC (phosphatidylcholine); DSPE (1,2 distearoyl-sn-glycero-3-phosphatidylethanolamine); PEG (polyethylene glycol); DOPE (dioleoyl phosphatidylethanolamine.

## Data Availability

Not applicable.
